# Case Report: Exacerbation and Spontaneous Separation of the Epiretinal Membrane Following Laser Photocoagulation of a Vasoproliferative Tumor of the Retina

**DOI:** 10.3389/fmed.2022.770689

**Published:** 2022-02-25

**Authors:** Luyao Tong, Yujie Jia, Junliang Wang, Yan Li, Zhiqing Chen, Shelan Liu, Li Zhang

**Affiliations:** ^1^Department of Ophthalmology, The Affiliated Hospital of Medical School of Ningbo University, Ningbo, China; ^2^Eye Center of Second Affiliated Hospital, School of Medicine, Zhejiang University, Hangzhou, China; ^3^Department of Ophthalmology and Otorhinolaryngology, Shaoxing Shangyu Women and Children Hospital, Shaoxing, China; ^4^Ningbo Eye Hospital, Ningbo, China; ^5^Hangzhou Linping Hospital of Integrated Traditional Chinese and Western Medicine, Hangzhou, China; ^6^Department of Infectious Diseases, Zhejiang Provincial Center for Disease Control and Prevention, Hangzhou, China

**Keywords:** vasoproliferative tumor of the retina, epiretinal membrane, laser photocoagulation, spontaneous separation, case report

## Abstract

The present report concerns a rare vasoproliferative tumor of the retina (VPTR) combined with a severe case of secondary epiretinal membrane (ERM). A 56-year-old male patient was diagnosed with VPTR and secondary ERM of the left eye. The patient underwent two rounds of laser photocoagulation (LP) of the tumor. The exacerbation of the ERM was observed after the first round of LP, while spontaneous separation over the five-month follow-up period was noted after the second round of LP. Thus, LP may represent a viable alternative treatment approach for VPTR combined with severe ERM.

## Introduction

A vasoproliferative tumor of the retina (VPTR), which is a rare kind of benign yellow or pink lesion composed of glial cells and vascular elements, is often associated with vision-threatening complications, including intraretinal exudation, exudative retinal detachment, pre-retinal or pre-macular fibrosis and macular edema (1–6). Satisfactory therapeutic effects in terms of vision improvement and lesion control have been reported in prior studies involving non-vitreoretinal surgical treatment, such as laser photocoagulation (LP), episcleral brachytherapy, intravitreal bevacizumab injection and cryotherapy. However, the formation of an epiretinal membrane (ERM) or macular hole and the exacerbation of vitreomacular traction have been identified as common complications of such treatments (4, 5, 7–11).

The effectiveness of vitrectomy in relation to VPTR combined with ERM has previously been described (6, 12, 13). Due to safety concerns, most ophthalmologists consider vitrectomy to be the treatment method of choice when an ERM is formed. However, a few cases of the spontaneous separation of the secondary ERM have been reported in patients with VPTR (14, 15). Surgical intervention could be avoided in approximately 3% of ERM cases (16).

Here, we report the case of a patient with VPTR who suffered severe ERM exacerbation following the first round of LP but experienced the spontaneous separation of the ERM after the second round of LP. To the best of our knowledge, in patients with VPTR, this is the first case of the exacerbation of ERM followed by spontaneous release to be reported.

## Case Report

**Figure 1:**
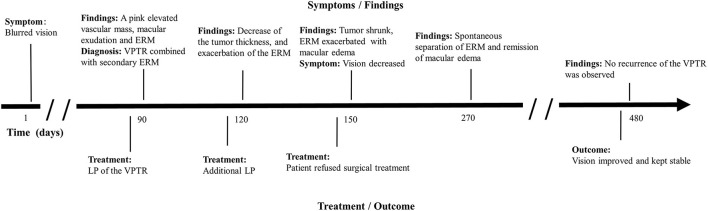
Clinical timeline for a 56-year-old Chinese male patient with a VPTR. VPTR, vasoproliferative tumor of the retina; ERM, epiretinal membrane; LP, laser photocoagulation.

**Figure 2:**
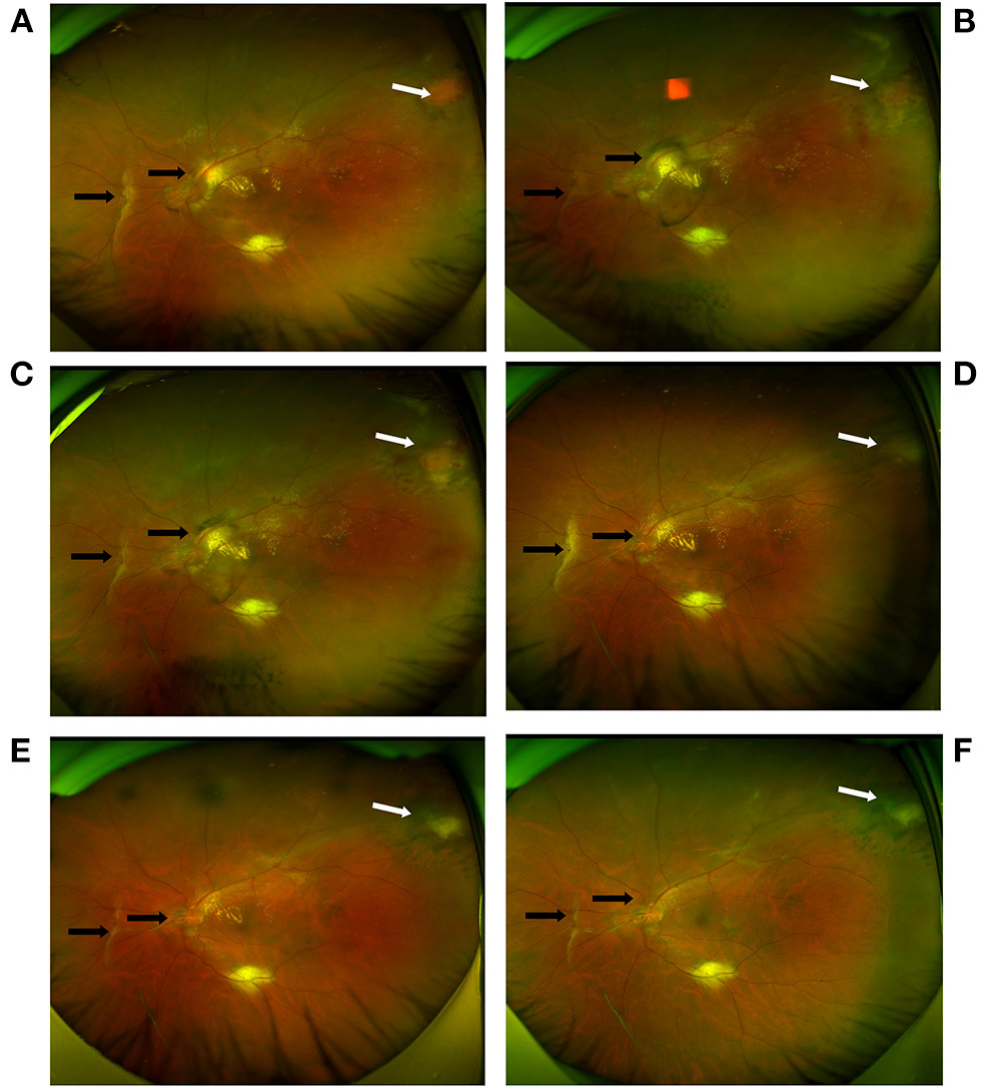
Ultra-widefield fundus photography imaging before and after LP in the left eye. **(A)** A pink elevated tumor located in the superotemporal retina (white arrow). Vitreoretinal and disc traction (black arrow), vitreous hemorrhage and macular exudation were also observed. **(B,C)** At the 2-week and 1-month follow-up appointments after the first round of LP, the tumor thickness decreased, although the tumor existed (white arrow). Vitreoretinal and disc traction persisted (black arrow). **(D–F)** At 1 month, 5 months and 1 year after the second round of LP, the tumor disappeared and did not recur (white arrow). Vitreoretinal traction gradually reduced (black arrow). Macular exudation gradually disappeared.

**Figure 3:**
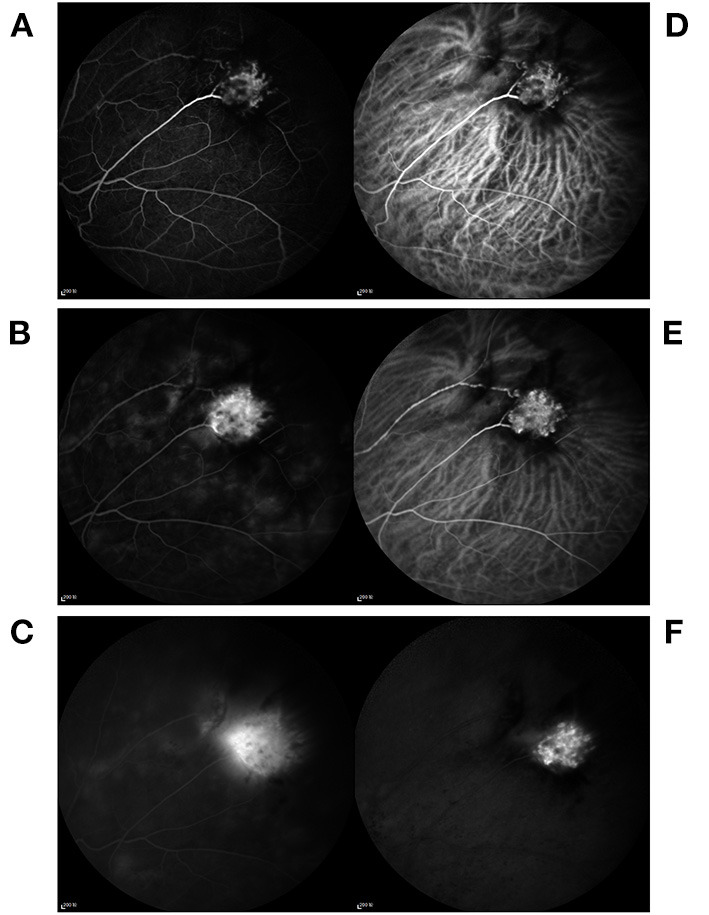
Fluorescein and indocyanine green angiography imaging in the left eye. **(A–C)** Fluorescein angiography showed the hyperfluorescence of the left eye mass to be at an early stage and the leakage to be at a late stage. **(D–F)** Indocyanine green angiography revealed the hypercyanescence to be at the middle and late stages.

A 56-year-old Chinese male patient was referred to our clinic due to a complaint of blurred vision in the left eye that had persisted for 3 months (Figure 1). He had no history of eye surgery or any infection, uveitis, trauma, or systemic disease. The patient's best-corrected visual acuity (BCVA) was 20/40 in the right eye and 20/200 in the left eye. Examination of the anterior segment revealed mild cortical cataracts in both eyes, while examination of the posterior segment showed a pink elevated mass approximately 5 mm in diameter located in the superotemporal retina of the left eye. The retinal “feeder” vessels of the mass exhibited a normal caliber without dilated or tortuous appearance. Slight vitreous hemorrhage, vitreous-disc traction, ERM and macular exudation were also observed in the patient's left eye (Figure 2). Moreover, a myelinated nerve fiber was identified in the inferior temporal region. The intraocular pressure was normal in both eyes. Fluorescein angiography (FA) revealed the hyperfluorescence of the mass to be at an early stage and the leakage of fluorescence to be at a late stage. Further, indocyanine green angiography (ICGA) indicated the hypercyanescence to be at the middle and late stages (Figure 3). A diagnosis of VPTR combined with secondary ERM in the patient's left eye was made.

**Figure 4:**
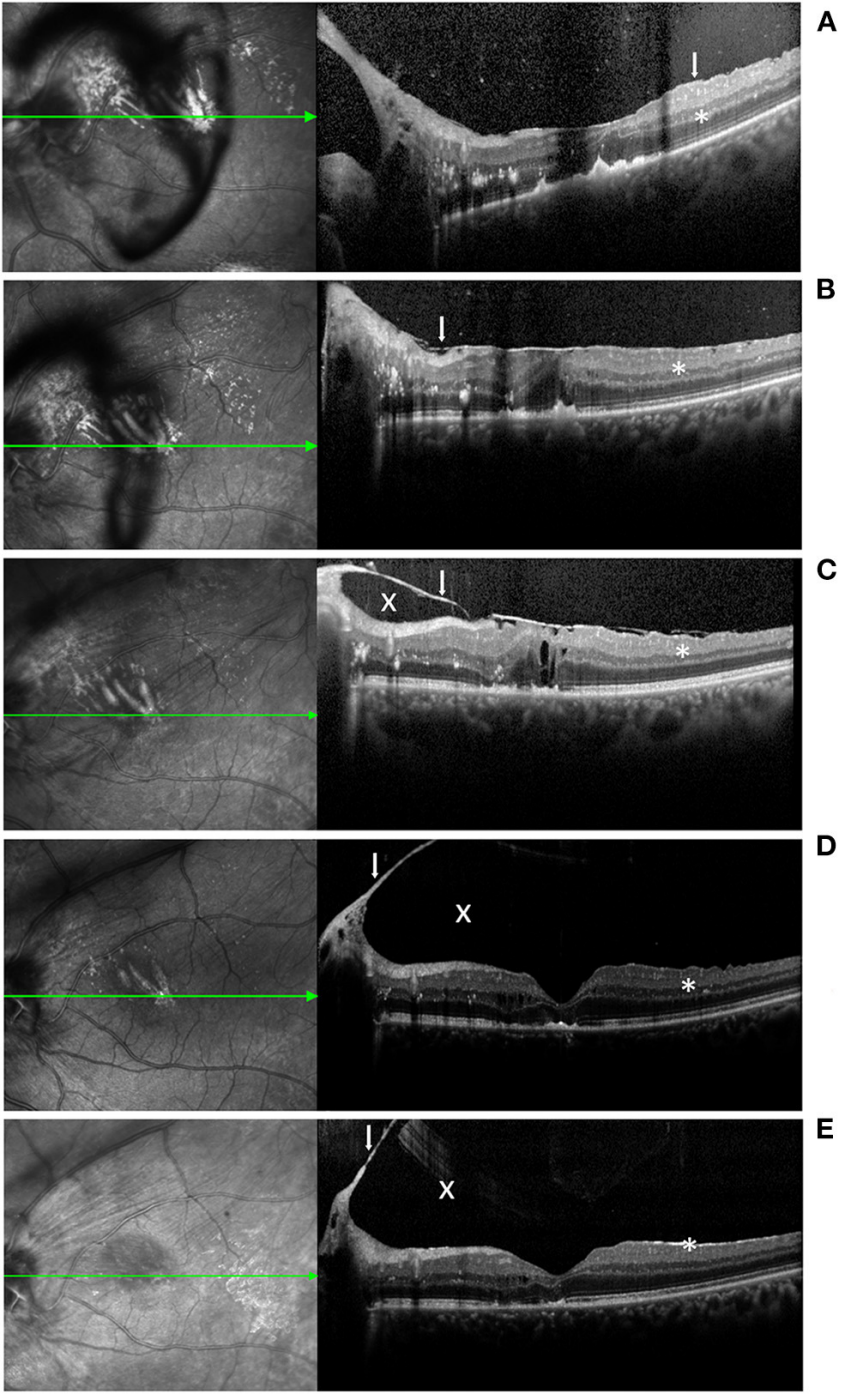
Macular OCT imaging before and after LP in the left eye. **(A,B)** At 2 weeks and 1 month after the first round of LP, ERM exacerbation (white arrow) and adherence to the retina (*) were observed. **(C)** At 1 month after the second round of LP, the ERM persisted and cystoid macular edema was observed. A cavity appeared between the ERM and retina. **(D,E)** At 5 months and 1 year after the second round of LP, the ERM separated spontaneously concomitant with PVD. The inner segment/outer segment layer was partially defected in the macular.

The patient underwent LP (Pascal Photocoagulator, Optimedica, Santa Clara, CA, USA; argon green laser [532 nm], duration of 0.2 s and spot size of 200 mm), which resulted in gray-white burns to the tumor in the left eye. At the 2- and 4-week follow-up appointments, the tumor thickness was found to have decreased, although the exacerbation of the ERM was observed by means of spectral domain optical coherence tomography (SD-OCT) (Spectralis OCT, Heidelberg, Germany). The BCVA of the patient's left eye remained 20/200. Due to the persistence of the mass, the patient underwent a second round of LP at the 4th week following the initial round of LP. The tumor was found to have shrunk one month after the second round of LP. Yet, the ERM persisted and cystoid macular edema was observed. Moreover, the patient's BCVA decreased to counting fingers. The patient refused further surgical treatment. At the fifth-month follow-up appointment after the second round of LP, the spontaneous separation of the ERM and the remission of the macular edema were observed by means of OCT. However, the inner segment/outer segment layer was found to be partially defective in the macular area. The patient's BCVA improved to 20/200. At the 12-month follow-up appointment after the second round of LP, no recurrence of the VPTR was observed. The relief of the vitreomacular traction was determined using OCT. The absorption of the exudation was observed by means of both OCT and fundus photography. The patient's BCVA was stable at 20/200. The ultra-widefield fundus photography and OCT images are available in Figures 2, 4, respectively.

## Discussion

Clinically, a VPTR is usually diagnosed on the basis of FA, ICGA, the tumor appearance and the morphology of the nourishing vessels. The most important differential diagnosis of VPTR is retinal capillary hemangioblastomas, which is the most common presenting feature of Von Hippel-Lindau's disease (17). Different from the unilateral eye and single lesion in this VPTR case, retinal capillary hemangioblastomas are usually bilateral and multiple. Most importantly, the feeding arteries and veins of the retinal capillary hemangioblastomas are very typically dilated (18). In our case, the VPTR was diagnosed on the basis of the FA and ICGA findings combined with the pink fundus mass and normal vessel findings. Prior studies involving patients with VPTR have achieved satisfactory therapeutic effects using cryotherapy (mean tumor diameter: 6 mm; tumor resolution: 16/16) (14), LP (maximum tumor diameters: 0.68–3.85 mm; tumor resolution: 8/8) (15) and episcleral brachytherapy (mean tumor diameter: 8.6 mm; tumor resolution: 29/30) (5). Photocoagulation has previously been reported to achieve the regression of the tumor in cases of VPTR (11, 15). A VPTR is generally associated with vision-threatening complications, especially ERM and macular edema (1, 5, 6). The incidence of secondary ERM has been found to range from 4.5 to 67% (1, 5, 6, 9, 19). In our case, the patient's VPTR persisted after the first round of LP and was accompanied by the exacerbation of the ERM. However, after the second round of LP, the VPTR regressed without recurrence and the ERM spontaneously separated. The case also demonstrated the valuable effect of LP in relation to VPTR. In this case, the patient's BCVA decreased following the ERM exacerbation and improved after the VPTR regression.

It has been suggested that the formation of a secondary ERM is related to the natural progression of VPTR (5, 11, 12, 14, 19). In fact, ERMs mostly originate from vitreous fibroblasts or retinal pigment epithelial cells. Pathological findings have suggested that the inner retinal layers and outer retinal surface are involved in VPTR (6). A previous study found that the secondary ERM identified in a VPTR case was mainly comprised of a hyaline membrane with positive staining for type IV collagen and glial fibrillary acidic protein, which was determined to be identical to the posterior hyaloid membrane (20). In addition, photocoagulation-related retinal gliosis and inflammation might increase the contractile activity of an ERM (15). Thus, we hypothesized that the secondary ERMs seen in some cases of VPTR might stem from a disturbed vitreous. If this is correct, the migrating vitreous cells would act as macrophages and stimulate further proliferation and fibrosis. However, further pathological studies are required to confirm our hypothesis.

In previous studies, vitrectomy has been used to peel the ERM (21). However, the spontaneous separation of the ERM was observed in 3% of the 1,248 tested eyes, which indicates that some patients could have avoided surgical intervention (16). Posterior vitreous detachment (PVD) is presumed to be an important pathological process related to spontaneous ERM separation (16), which may be associated with LP (15) and cryotherapy (14). In our case, the OCT conducted before the second round of LP showed no signs of PVD, while the ERM was shown to be tightly adhered to the retina. After two rounds of LP, the OCT findings revealed the spontaneous separation of the ERM concomitant with PVD, which suggests that PVD is involved in the pathological process associated with spontaneous ERM separation. Interestingly, we observed the exacerbation of the secondary ERM after the first round of LP, which severely affected both the macula and the optic disc, rather than a trend toward regression. The ERM spontaneously separated in this case, thereby suggesting a different possibility, namely that the spontaneous separation of the secondary ERM in the VPTR eye may have resulted from a pathologic process of its own, rather than being a therapeutic effect of LP. Therefore, ophthalmologists should focus on treating the VPTR and closely following up on any morphological changes to the ERM. Although the presence of an ERM increases the risk of retinal detachment and macular hole (10), LP treatment for VPTR with secondary ERM remains a viable alternative approach. To investigate the efficacy and safety of LP as a non-operative treatment in relation to both VPTR and VPTR combined secondary ERM, long-term follow-up and case-series studies are needed.

## Conclusion

Severe secondary ERM may occur in cases of VPTR before or after LP treatment. If the affected eye has not yet developed PVD, LP may represent a viable alternative treatment approach for VPTR with severe secondary ERM.

## Data Availability Statement

The original contributions presented in the study are included in the article/Supplementary Material, further inquiries can be directed to the corresponding author/s.

## Ethics Statement

The studies involving human participants were reviewed and approved by Institutional Review Board of the Second Affiliated Hospital, School of Medicine, Zhejiang University(NO:2021-0292). The patients/participants provided their written informed consent to participate in this study. Written informed consent was obtained from the individual(s) for the publication of any potentially identifiable images or data included in this article.

## Author Contributions

LZ and SL designed the report. LZ and ZC followed-up the patient. LT and YJ wrote the manuscript. YL and JW collected the patient's clinical data. All authors read and approved the final manuscript.

## Conflict of Interest

The authors declare that the research was conducted in the absence of any commercial or financial relationships that could be construed as a potential conflict of interest.

## Publisher's Note

All claims expressed in this article are solely those of the authors and do not necessarily represent those of their affiliated organizations, or those of the publisher, the editors and the reviewers. Any product that may be evaluated in this article, or claim that may be made by its manufacturer, is not guaranteed or endorsed by the publisher.

## References

[1] Shields CL. Vasoproliferative tumors of the ocular fundus. Archiv Ophthalmol. (1995) 113:615. 10.1001/archopht.1995.01100050083035 7748132

[2] Heimann H. Vasoproliferative tumours of the retina. Br J Ophthalmol. (2000) 84:1162–9. 10.1136/bjo.84.10.1162 11004104 PMC1723269

[3] Irvine F. Retinal vasoproliferative tumors: surgical management and histological findings. Archiv Ophthalmol. (2000) 118:563. 10.1001/archopht.118.4.563 10766145

[4] Jain K Berger AR Yucil YH McGowan HD. Vasoproliferative tumours of the retina. Eye. (2003) 17:364–8. 10.1038/sj.eye.6700311 12724700

[5] Cohen VM Shields CL Demirci H Shields JA. Iodine I 125 plaque radiotherapy for vasoproliferative tumors of the retina in 30 eyes. Arch Ophthalmol. (2008) 126:1245–51. 10.1001/archopht.126.9.1245 18779485

[6] Zheng B Chen Y Chen L Chen H Zheng J Chen F. Comparative study on the efficacy and safety of tumor resection in vitrectomy for retinal vasoproliferative tumors. J Ophthalmol. (2019) 2019:7464123. 10.1155/2019/7464123 30719338 PMC6334322

[7] Castro-Navarro V Saktanasate J Say EA Chiang A Shields CL. Role of pars plana vitrectomy and membrane peel in vitreomacular traction associated with retinal vasoproliferative tumors. Oman J Ophthalmol. (2016) 9:167–9. 10.4103/0974-620X.192280 27843233 PMC5084501

[8] Marcelo-Tan MC Parsons HM. Retinal vasoproliferative tumour in an adolescent who had medulloblastoma: a case report. Canad J Ophthalmol J Canadien D'ophtalmologie. (2015) 50:e74–8. 10.1016/j.jcjo.2015.05.017 26455986

[9] Brockmann C Rehak M Heufelder J Cordini D Brockmann T Corkhill C. Predictors Of treatment response of vasoproliferative retinal tumors to ruthenium-106 brachytherapy. Retina. (2016) 36:2384–90. 10.1097/IAE.0000000000001095 27322946

[10] Sato A Fukui E Ohta K. Macular hole formation in eye after cryotherapy and intravitreal bevacizumab treatment for vasoproliferative tumor. Int Med Case Rep J. (2020) 13:419–23. 10.2147/IMCRJ.S266986 32982482 PMC7501591

[11] Krivosic V Massin P Desjardins L Le Hoang P Tadayoni R Gaudric A. Management of idiopathic retinal vasoproliferative tumors by slit-lamp laser or endolaser photocoagulation. Am J Ophthalmol. (2014) 158:154–61 e1. 10.1016/j.ajo.2014.03.005 24631475

[12] Garcia-Arumi J Distefano LN Fonollosa A Quijano C Corcostegui B. Management of vision-threatening complications of vasoproliferative tumors of the retina. Ophthalmic Res. (2015) 54:34–40. 10.1159/000430955 26065358

[13] Saito W Kase S Fujiya A Dong Z Noda K Ishida S. Expression of vascular endothelial growth factor and intravitreal anti-vegf therapy with bevacizumab in vasoproliferative retinal tumors. Retina. (2013) 33:1959–67. 10.1097/IAE.0b013e3182923490 23652580

[14] Manjandavida FP Shields CL Kaliki S Shields JA. Cryotherapy-induced release of epiretinal membrane associated with retinal vasoproliferative tumor: analysis of 16 cases. Retina. (2014) 34:1644–50. 10.1097/IAE.0000000000000137 24752009

[15] Ding X Guo J Xu G Liu W. Photocoagulation-associated spontaneous release of epiretinal membrane secondary to retinal vascular tumor: case series of 8 cases. Lasers Med Sci. (2021) 6:18. 10.1007/s10103-021-03351-9 34138385

[16] Yang HS Hong JW Kim YJ Kim J-G Joe SG. Characteristics of spontaneous idiopathic epiretinal membrane separation in spectral domain optical coherence tomography. Retina. (2014) 34:2079–87. 10.1097/IAE.0000000000000199 24830825

[17] Christoferson LA Gustafson MB Petersen AG. Von hippel-lindau's disease. JAMA. (1961) 178:280–2. 10.1001/jama.1961.03040420020005 13879311

[18] Karacorlu M Hocaoglu M Sayman Muslubas I Ersoz MG Arf S. Therapeutic outcomes after endoresection of complex retinal capillary hemangioblastoma. Retina. (2018) 38:569–77. 10.1097/IAE.0000000000001562 28196061

[19] Abolfathzadeh N Naseripour M Jaberi R Azma Z Alemzadeh SA Arbab M. Ru-106 Plaque radiotherapy for vasoproliferative tumors of retina: a 15-year single-center experience. Int Ophthalmol. (2020) 40:2095–102. 10.1007/s10792-020-01386-5 32361860

[20] Shankar P Bradshaw SE Ang A Rennie IG Snead DR Snead MP. Vascularised epiretinal membrane associated with vasoproliferative tumour. Eye. (2007) 21:1003–4. 10.1038/sj.eye.6702800 17417621

[21] Khan MA Kuley A Riemann CD Berrocal MH Lakhanpal RR Hsu J. Long-term visual outcomes and safety profile of 27-gauge pars plana vitrectomy for posterior segment disease. Ophthalmology. (2018) 125:423–31.. 10.1016/j.ophtha.2017.09.013 29146307

